# Supplementary material for: Multi‐dataset identification of innovative feature genes and molecular mechanisms in keratoconus
Source: J Cell Mol Med. 2024 Sep 19;28(18):e70079. doi: 10.1111/jcmm.70079 (PMC11412914; doi:10.1111/jcmm.70079)
Supplement: Supplementary file 2 — Material S2. [file JCMM-28-e70079-s002.zip › 7.GSEA analysis/3.DBNDD1/KEGG_OTHER_GLYCAN_DEGRADATION.html]

Details for gene set KEGG\_OTHER\_GLYCAN\_DEGRADATION[GSEA]

|  || Dataset | DBNDD1.group.cls#h\_versus\_l |
| Phenotype | group.cls#h\_versus\_l |
| Upregulated in class | l |
| GeneSet | KEGG\_OTHER\_GLYCAN\_DEGRADATION |
| Enrichment Score (ES) | -0.45124236 |
| Normalized Enrichment Score (NES) | -1.2823238 |
| Nominal p-value | 0.20833333 |
| FDR q-value | 0.7262145 |
| FWER p-Value | 0.969 |
Table: GSEA Results Summary

  

Fig 1: Enrichment plot: KEGG\_OTHER\_GLYCAN\_DEGRADATION      
 Profile of the Running ES Score & Positions of GeneSet Members on the Rank Ordered List

  

| SYMBOL | TITLE | RANK IN GENE LIST | RANK METRIC SCORE | RUNNING ES | CORE ENRICHMENT || 1 | FUCA1 | na | 10096 | 0.070 | -0.2160 | No |
| 2 | MAN2B2 | na | 10104 | 0.070 | -0.1415 | No |
| 3 | NEU3 | na | 10403 | 0.066 | -0.0800 | No |
| 4 | NEU4 | na | 11727 | 0.050 | -0.0651 | No |
| 5 | HEXA | na | 13363 | 0.035 | -0.0746 | No |
| 6 | FUCA2 | na | 13887 | 0.031 | -0.0567 | No |
| 7 | AGA | na | 17051 | 0.009 | -0.1382 | No |
| 8 | GLB1 | na | 18534 | -0.001 | -0.1797 | No |
| 9 | ENGASE | na | 19131 | -0.005 | -0.1911 | No |
| 10 | MAN2C1 | na | 20720 | -0.019 | -0.2162 | Yes |
| 11 | MAN2B1 | na | 21008 | -0.022 | -0.2008 | Yes |
| 12 | NEU1 | na | 21377 | -0.026 | -0.1841 | Yes |
| 13 | HEXB | na | 21627 | -0.028 | -0.1612 | Yes |
| 14 | MANBA | na | 24582 | -0.065 | -0.1775 | Yes |
| 15 | NEU2 | na | 34086 | -0.441 | 0.0185 | Yes |
Table: GSEA details [plain text format]

  

Fig 2: KEGG\_OTHER\_GLYCAN\_DEGRADATION      
 Blue-Pink O' Gram in the Space of the Analyzed GeneSet

  

Fig 3: KEGG\_OTHER\_GLYCAN\_DEGRADATION: Random ES distribution      
 Gene set null distribution of ES for **KEGG\_OTHER\_GLYCAN\_DEGRADATION**
